# Systematic pan‐cancer analysis identifies RBM39 as an immunological and prognostic biomarker

**DOI:** 10.1111/jcmm.17517

**Published:** 2022-08-21

**Authors:** Rui Zhang, Wei Wang, Nie Zhang, Xueting Chen, Wanming Liu, Longzhen Zhang, Nianli Liu

**Affiliations:** ^1^ Cancer Institute Xuzhou Medical University Xuzhou China; ^2^ Department of Radiation Oncology Affiliated Hospital of Xuzhou Medical University Xuzhou China; ^3^ Center of Clinical Oncology Affiliated Hospital of Xuzhou Medical University Xuzhou China

**Keywords:** cancer immunology, immunological biomarker, pan‐cancer, prognostic marker, RBM39

## Abstract

RNA‐binding Motif Protein39 (RBM39) is identified as a splicing factor and transcription coactivator. Despite mounting evidence that RBM39 plays a critical role in the development of specific malignancies, no systematic pan‐cancer investigation of RBM39 has been conducted. As a result, we set out to investigate RBM39’s prognostic significance and putative immunological activities in 33 different cancers. Based on TCGA and CCLE, GTEx, cBioportal and HPA, we used a series of bioinformatics approaches to explore the potential oncogenic role of RBM39, including analysis of the expression of the pan‐cancer species RBM39, the prognostic relationship between RBM39 expression and overall survival (OS), disease‐specific survival (DSS) and progression‐free interval (PFI), the relationship between RBM39 expression and clinical phenotype, analysis of the relationship between RBM39 expression and tumour mutational burden (TMB), microsatellite instability (MSI), DNA methylation and immune cell infiltration. Our results showed that RBM39 is overexpressed in most cancers. RBM39 was positively or negatively correlated with the prognosis of different tumours. RBM39 expression was associated with TMB and MSI in 9 and 12 cancer types. In addition, RBM39 expression was associated with DNA methylation in almost all tumours. There are eight tumours were screened for further study, including BRCA, COAD, HNSC, LIHC, LUSC, SKCM, STAD, UCEC. In the screed tumours, RBM39 was found to be negatively correlated with the infiltration of most immune cells. In addition, the correlation with RBM39 expression varied by immune cell subtype. Based on RBM39’s role in tumorigenesis and tumour immunity, we suggest it can serve as a surrogate prognostic marker.

## 1 INTRODUCTION

Cancer, as a highly complex disease, is the leading cause of death in every country in the world and an important obstacle to prolonging life expectancy. By 2020, it is estimated that there will be nearly 10 million cancer deaths and 19.3 million new cancer cases worldwide.^1^ Recently, tumour immunotherapy has become a prominent cancer treatment approach. As public databases, like TCGA and CCLE, continue to develop and improve, new immunotherapeutic targets can be identified by performing pan‐cancer expression analyses of genes and evaluating their correlation with clinical characteristics.

RNA‐binding motif (RBM) proteins are members of the family of RNA‐binding proteins (RBPs), which are present in all organisms and can bind single‐ or double‐stranded RNA and play important roles as post‐transcriptional regulators in regulating RNA metabolism and gene expression.^2^ By binding RNA, RBPs aggregate in ribonucleoprotein complexes that determine the destiny and function of almost every cellular RNA molecule.^3^, ^4^ This protein, also known as RBM39, HCC1, Caperα, FSAP59 and RNPC2, was found to be distributed in a very speckled network within the nucleus and to colocalize with SC35 and uridine‐rich small nuclear RNA.^5^ RBM39 is involved in transcriptional regulation and RNA splicing. Selective splicing has been shown to be a widespread phenomenon that promotes the multiplication of gene products in a tissue‐specific manner.^6^, ^7^ Recent findings reveal diverse ways in which splicing is pathologically changed to facilitate the initiation and/or retention of cancer.^8^It contains a trans‐activating structural domain, a serine‐arginine‐rich pair region and an RNA recognition motif, which consists of two highly conserved peptide motifs with RNA and single‐stranded cDNA binding activity.^9^ It has been shown that RBM39, a transcriptional coactivator of activator protein‐1 (AP‐1/Jun), oestrogen receptor and NF‐kB,^10^, ^11^, ^12^ is involved in energy and redox homeostasis and cancer cell proliferation, in addition to its role in vascular endothelial growth factor splicing.^13^ Some studies have demonstrated that RBM39 is a proto‐oncogene that plays an important role in the development of several malignancies. In many preclinical models, loss of RBM39 leads to aberrant splicing events and differential gene expression, which inhibit cell cycle progression and lead to tumour regression.

So far, most studies on the role of RBM39 in tumours have been limited to one specific type of cancer. There are no pan‐cancer studies on the relationship between RBM39 and various cancers. Therefore, we analysed the expression levels of RBM39 in different types of malignancies and its relationship with prognosis using multiple databases, including TCGA, Cancer Cell Line Encyclopedia (CCLE), Genotype‐Tissue Expression (GTEx), cBioPortal and Human Protein Atlas (HPA). In addition, we explored the relationship of RBM39 expression with DNA methylation and immune infiltration. We introduced co‐expression analysis and enrichment analysis of genes to investigate the biological functions of RBM39 in tumours. Our results suggest that RBM39 may play an important role in tumours and serve as a prognostic factor. Our study provides a theoretical basis to gain insight into the role of RBM39 in tumour immunotherapy.

## 2 METHODS

### 2.1 Data processing and differential expression analysis

RNA sequencing, somatic mutations and clinical data were gathered from the TCGA and GTEx databases. Each tumour cell line's CCLE‐based expression data was obtained from the Broad Institute's Portal (https://portals.broadinstitute.org/). EdgeR software was used to compare RBM39 expression levels in tumour and normal tissues. Using the Kruskal–Wallis test, we determined the levels of RBM39 expression in several normal tissues and tumour cell lines.

### 2.2 Immunohistochemical staining

IHC results of RBM39 protein expression were collected from the HPA database (https://www.proteinatlas.org/) to compare differences in RBM39 protein expression between tumour tissues and normal tissues, including breast invasive carcinoma (BRCA), liver hepatocellular cancer (LIHC), lung adenocarcinoma (LUAD), lung squamous carcinoma (LUSC) and colon adenocarcinoma (COAD).

### 2.3 Analysis of the connection between RBM39 and prognosis and clinical phenotype

TCGA data on each sample's survival and clinical features were obtained. Three indicators, OS, DSS and PFI, were considered when analysing the correlation between RBM39 and survival. Kaplan–Meier (*p* < 0.05) survival analysis was used. Age, tumour stage and clinical phenotypes were analysed to see whether they were associated with RBM39 expression (p < 0.05).

### 2.4 Correlation analysis of RBM39 with the tumour mutational burden (TMB) and microsatellite instability (MSI)

For the purpose of calculating TMB for each tumour specimen independently, Spearman rank correlation coefficient was utilized. TMB is a biomarker that reflects mutations of tumour cells. A microsatellite instability (MSI) occurs when an insertion or deletion of repetitive units results in a change in microsatellite length as compared with normal tissues. Spearman rank correlation coefficient was used to analyse the correlation between RBM39 expression and MSI.

### 2.5 Correlation analysis of RBM39 with DNA Mismatch Repair Genes and Methyltransferases

Mismatch repair is an intracellular process that allows mismatches to be repaired. DNA replication errors are not repaired when this mechanism fails, resulting in an increase in somatic mutations. The relationship between five of the MMRS genes (MLH1, MSH2, MSH6, PMS2, EPCAM) and RBM39 expression was studied using the TCGA data. The chemical modification of DNA known as DNA methylation can affect epigenetics and can influence gene expression without changing the DNA sequence. We used this study to examine whether RBM39 expression correlated with that of the four methyltransferases.

### 2.6 Correlation analysis of RBM39 with the immune microenvironment

Tumour‐infiltrating lymphocytes are independently correlated with the status of primary lymph nodes and survival, and tumour immune cell scores are determined using immune scores and interstitial scores. We used software estimation to analyse the correlation between gene expression and immune cell score.

### 2.7 Correlation analysis of RBM39 with immune characteristics

A neoantigen is encoded by a modified gene in tumour cells as a result of biological processes like point mutations, removal mutations and mutation fusions. Scanner calculates its binding affinity score based on antigenic epitopes with a length of 8–11 amino acids, whereas neoantigens have a score less than 500 nm. The Scanner software was used to quantify the number of neoantigens in each tumour specimen independently, as well as to examine RBM39 expression in relation to the number of antigens. In addition, 47 frequently studied immune checkpoint genes were isolated independently from RBM39 expression, and correlations with RBM39 expression were calculated.

We used CIBERSORT, a metagene tool that predicts immune cell phenotypes, to assess the correlations between RBM39 levels and immune cell infiltration in cancer in 33 tumours. (*p* < 0.05 was considered significant).

Furthermore, we looked at the co‐expression of RBM39 and immune‐related genes, such as those encoding major histocompatibility complex (MHC), immune activation, immunosuppression, chemokine and chemokine receptor proteins. Gene Set Enrichment Analysis of RBM39 across cancers. The biological activities of RBM39 in malignancies were investigated using Gene Set Enrichment Analysis (GSEA).

## 3 RESULTS

### 3.1 Expression of RBM39 in pan‐cancer

**FIGURE 1:**
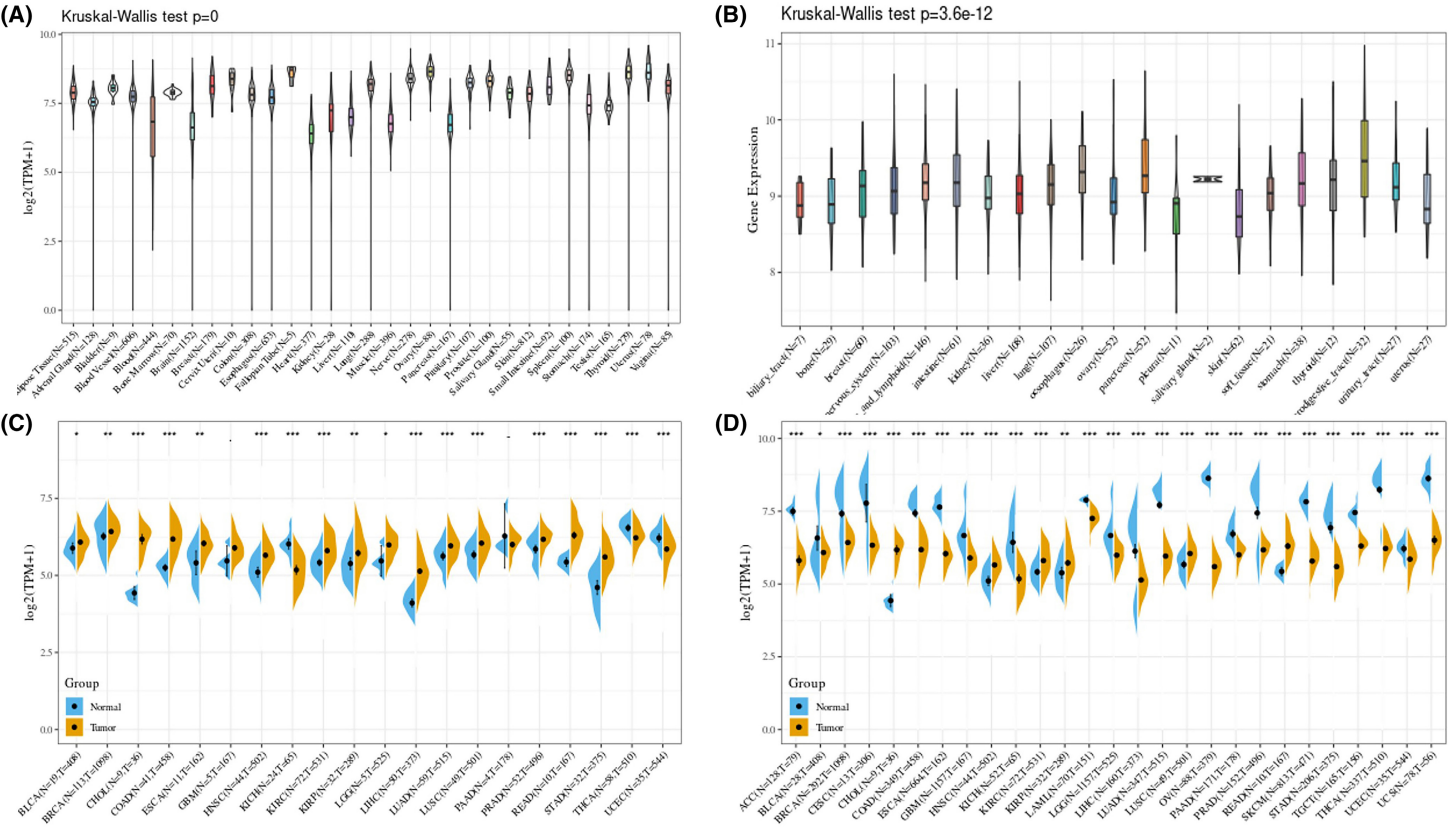
RBM39 expression across cancers. (A) RBM39 expression levels in a sample of 31 tissues collected from the GTEx. (B) RBM39 expression levels in a dataset produced from the CCLE including 21 tissues in tumour cell lines. (C) RBM39 expression levels in tumour and matched neighbouring noncancerous tissues from the TCGA, *p 0.05, **p 0.01, ***p 0.001. (D) RBM39 expression differential in 27 cancers combining GTEx normal tissue data and TCGA tumour tissue data, *p < 0.05, **p < 0.01, ***p < 0.001

We analysed the expression levels of the physiological RBM39 gene across tissues through the GTEx database (Figure 1A). RBM39 was expressed at the highest and lowest levels in the oviductal and heart, respectively. Figure 1B depicts the relative RBM39 expression levels across various cell lines based on CCLE data. Then, we used the TCGA database to obtain the differential expression patterns of RBM39 in cancer and paraneoplastic tissue samples (Figure 1C). It can be noted that most tumour tissues have greater RBM39 expression than surrounding normal tissues. We investigated changes in RBM39 expression in 27 tumour tissues by merging data from GTEx normal tissues and TCGA tumour tissues (Figure 1D). From the analysis of RBM39 expression in pan‐cancer, it was found that RBM39 showed high expression in six tumours, including cholangiocarcinoma (CHOL), rectum adenocarcinoma (READ), head and neck squamous cell carcinoma (HNSC), lung squamous cell carcinoma (LUSC), kidney clear cell carcinoma (KIRC) and kidney papillary cell carcinoma (KIRP). While in the remaining 19 tumours, the expression level of RBM39 in tumours was generally lower than that in normal tissues.

Furthermore, to assess RBM39 protein expression, we matched IHC results from the HPA database to RBM39 gene expression data from the TCGA database. The results showed that normal breast, lung, liver and colon tissues showed low levels of RBM39 staining, whereas the corresponding breast, lung squamous, liver and colon malignancies had moderate to high expression, which was consistent with the expression pattern of RBM39 in the TCGA database for the five tumours (Figure S1A–E).

### 3.2 Prognostic value of RBM39 across cancers

**FIGURE 2:**
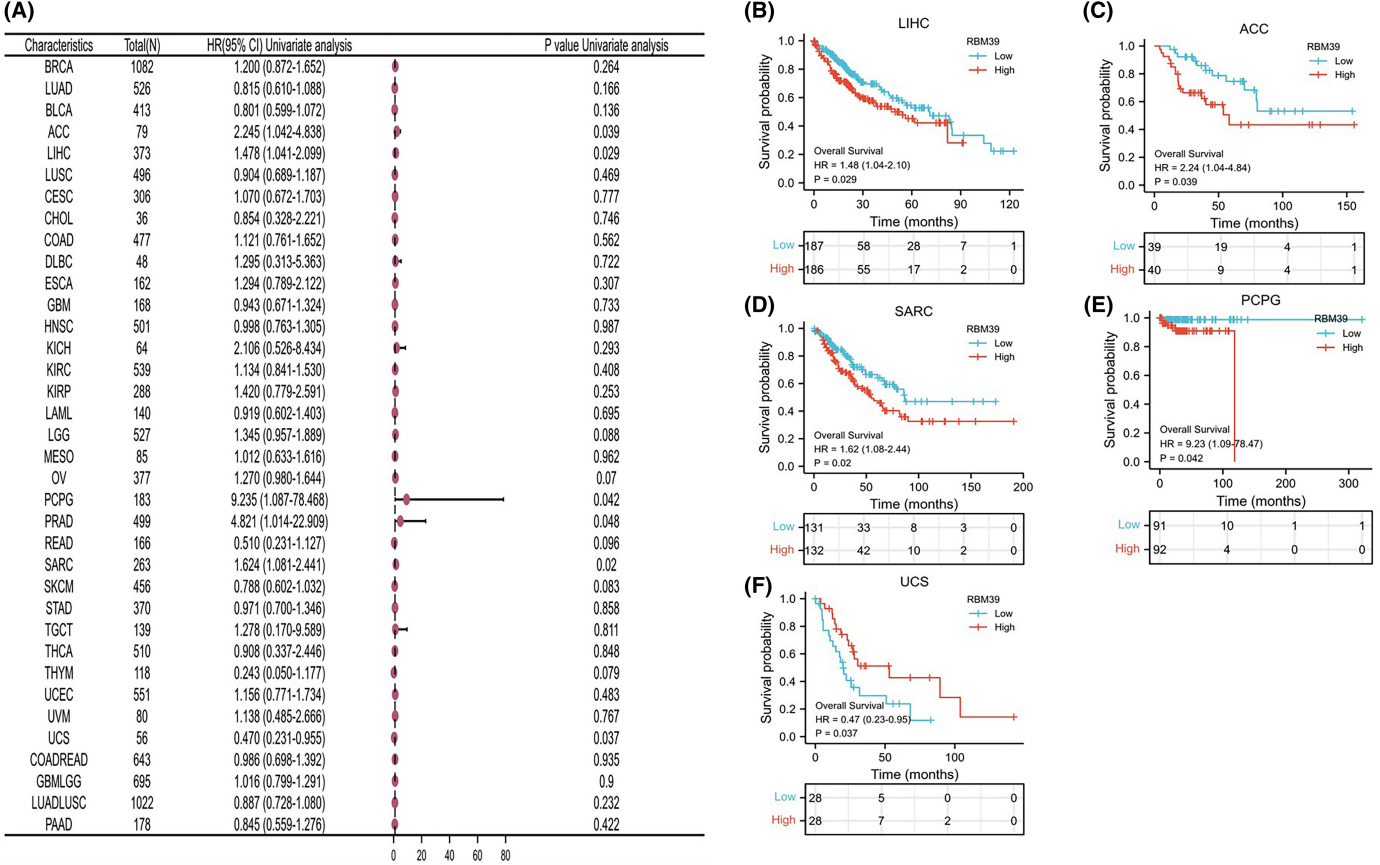
RBM39 expression and total survival time in days are related (OS). (A) Tumour OS association forest plot. (B–F) A Kaplan–Meier analysis of the relationship between RBM39 expression and OS is shown

To assess the link between RBM39 expression levels and prognosis, we ran a survival association study for each cancer, including OS, DSS and PFI. As shown in Figure 2A, COX proportional hazards risk model analysis showed that RBM39 expression levels were associated with OS in LIHC (HR = 1.48, *p* = 0.029), ACC (HR = 2.25, *p* = 0.039), SARC (HR = 1.62, *p* = 0.02), UCS (HR = 0.47, *p* = 0.037), PRAD (HR = 4.82, *p* = 0.048), PCPG (HR = 9.24, *p* = 0.042). These results suggested that RBM39 was a high‐risk gene in LIHC, ACC, SARC, PRAD and PCPG, while it was a low‐risk gene in UCS. A Kaplan–Meier survival study revealed that patients with low RBM39 expression in LIHC, ACC, SARC, PRAD and PCPG had a longer OS, whereas UCS patients with low RBM39 expression had a shorter overall survival (Figure 2B–F).

In addition, analysis of DSS data (Figure S2A) showed that low expression of RBM39 was associated with poor prognosis in patients with LIHC (HR = 1.718, *p* = 0.019) and SARC (HR = 1.636, *p* = 0.032). Kaplan–Meier survival analysis showed that patients with low RBM39 expression in both tumours had longer disease‐specific survival (Figure S2B,C). In terms of the relationship between RBM39 expression and PFI, forest plots revealed a link between high expression and low PFI in LIHC (HR = 1.34, *p* = 0.046), ACC (HR = 2.03, *p* = 0.028), UCEC (HR = 1.61, *p* = 0.008), PRAD (HR = 1.84, *p* = 0.004). However, high expression of RBM39 was associated with high PFI in SKCM (HR = 0.75, *p* = 0.011) (Figure S3A). KM survival analysis showed that LIHC, ACC, UCEC, PRAD and SKCM patients with high RBM39 expression had lower survival, while SKCM patients with high RBM39 expression had longer survival (Figure S3B–F).

### 3.3 Relationship between RBM39 expression and clinical phenotypes

Next, we investigated how RBM39 expression differed by age in patients with different tumour types. We found that patients aged >60 years with HNSC (*p* < 0.01), KIRP (*p* < 0.01) and SARC (*p* = 0.01) had lower RBM39 expression, while UCEC (*p* = 0.02) patients <60 years had higher RBM39 expression (Figure S4A–D). We also looked at the association between RBM39 expression and tumour stage. RBM39 expression was shown to be substantially linked with tumour stage in four malignancies studied (Figure S4E–H), including LIHC, KICH, THCA and KIRC. Significant differences in RBM39 expression occurred between stage I and II tumours in KICH and THCA were observed. Interestingly, in contrast to LIHC and KIRC, whose RBM39 expression increased significantly with increasing tumour stage between stage I and III, most other types of cancer did not show a statistically significant difference in terms of tumour stage.

### 3.4 Expression of RBM39 in pan‐cancerous tissues correlates with TMB and MSI

**FIGURE 3:**
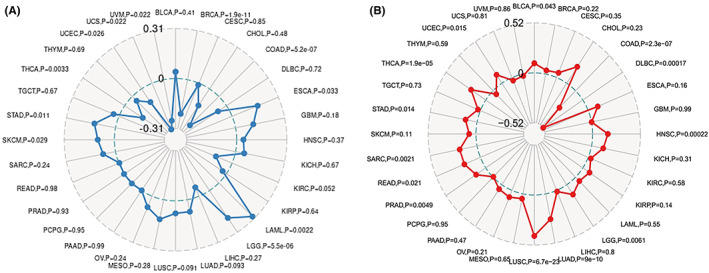
Correlation investigation of RBM39 expression in pan‐cancer, TMB, and MSI. (A) Correlation study of RBM39 expression in pan‐cancer and TMB. (B) Explained are the findings of a correlation analysis between RBM39 expression in pan‐cancer and MSI

Tumour mutational burden is typically defined as the total amount of nonsynonymous mutations occurring on an average of 1 M bases in the tumour cell genome's coding region, and the mutation types primarily comprise single nucleotide variants (SNVs) and minor insertions/deletions (INDELs). TMB is a quantifiable biomarker that indicates the number of mutations discovered in tumour cells. As shown in Figure 3A, the association between TMB and RBM39 expression was statistically examined using Spearman rank correlation coefficients for each tumour type independently. Notably, RBM39 expression was positively correlated with TMB in ESCA, LAML, LGG, SKCM and STAD, and negatively correlated with UCS, UVM, UCEC, BRCA, THCA and COAD. MSI is a term that describes any change in microsatellite length caused by the insertion or deletion of repeat units in tumours compared with normal tissue, as well as the introduction of novel microsatellite alleles. As shown in Figure 3B, the correlation between RBM39 and MSI was analysed using the Spearman rank correlation coefficient. The results showed that RBM39 was positively correlated with MSI in HNSC, LGG, LUAD, LUSC, PRAD, READ, STAD, THCA, UCEC and BLCA, and negatively correlated with DLBC and COAD.

### 3.5 Correlation of RBM39 expression with DNA mismatch repair genes and methyltransferases in pan‐cancer

With the exception of CHOL, practically all MMRs genes were related with RBM39 expression across cancers, suggesting that RBM39 may protect tumour cell survival by promoting DNA mismatch repair‐related genes expression (Figure S5A). DNA methylation is the action of DNA methyltransferases that covalently attach a methyl group to the 5′ carbon position of cytosine in genomic CpG dinucleotides. We discovered that the expression levels of RBM39 in all tumours were positively correlated with the expression levels of methyltransferases, as shown in Figure S5B, implying that RBM39 may play a role in carcinogenesis and progression by modulating the epigenetic status of human pan‐cancer.

### 3.6 Relationship between RBM39 and tumour microenvironment

**FIGURE 4:**
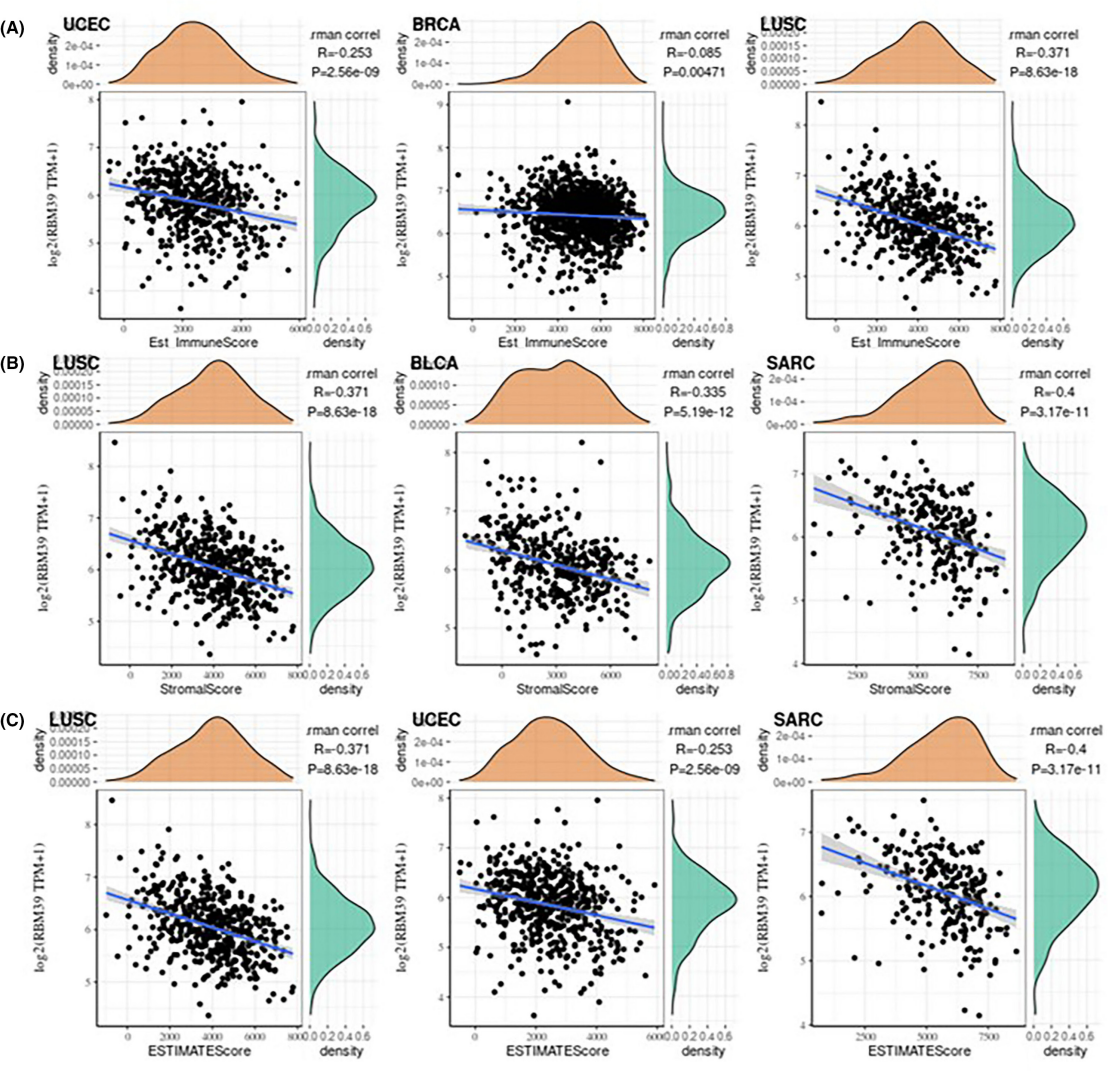
Correlation analysis between RBM39 expression in Pan‐cancer and immune score (A), stromal score (B) and estimate immune score (C)

We calculated the immunological score and interstitial score of individual tumour samples to see how RBM39 affects the tumour immune microenvironment throughout tumour progression. Among the 33 tumours, the top two tumours with the most significant correlation between RBM39 expression and immune score were UCEC (*R* = –0.253, *p* < 0.001) and LUSC (*R* = –0.371, *p* < 0.001), and the top three tumours whose RBM39 expression was most significantly correlated with mesenchymal scores were LUSC (*R* = –0.371, *p* < 0.001), BLCA (R = –0.335, *p* < 0.001) and SARC (*R* = –0.4, *p* < 0.001); the top three tumours with the highest correlation with the assessed immune scores were LUSC (*R* = –0.371, *p* < 0.001), UCEC (*R* = –0.253, *p* < 0.001) and SARC (*R* = –0.4, *p* < 0.001) (Figure 4A–C). The findings revealed that RBM39 expression levels in UCEC, BRCA and LUSC were substantially and adversely connected with the immunological score in the tumour immune microenvironment, and that RBM39 expression levels in LUSC, UCEC and SARC were also negatively correlated with the interstitial score.

### 3.7 Expression of RBM39 in pan‐cancer is correlated with immune neoantigens and immune checkpoint genes

**FIGURE 5:**
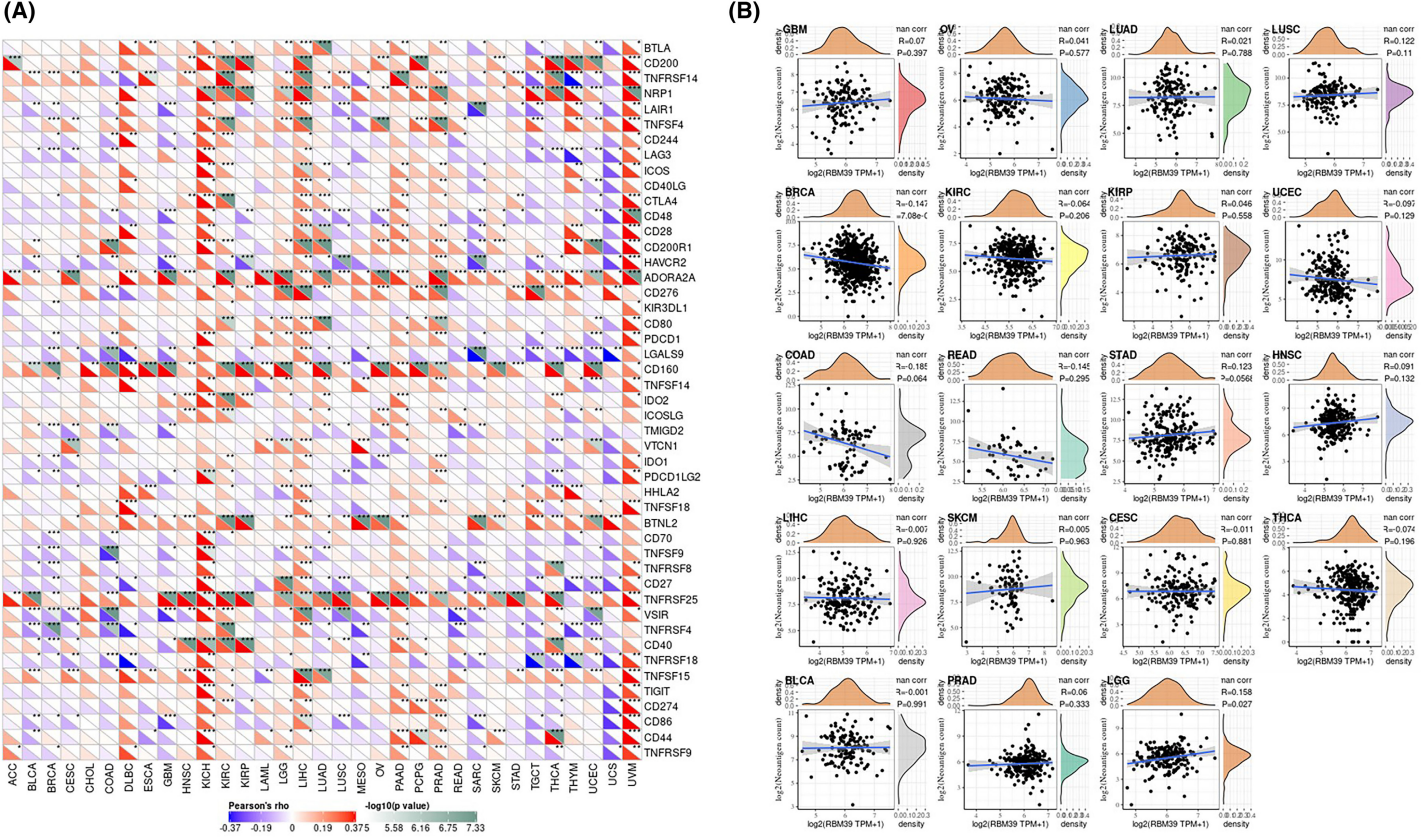
RBM39 expression, immunological checkpoint genes, and immune neoantigens in pan‐cancer correlation analysis. (A) Analysis of the relationship between RBM39 expression and immune checkpoint gene expression in pan‐cancer patients. (B) A correlation study of RBM39 expression in pan‐cancer and the amount of tumour neoantigens in 19 different tumour types was performed

We next examined the correlation between RBM39 with more than 40 immune checkpoint genes across cancers. RBM39 expression was shown to be favourably linked with immune checkpoint gene expression levels in a variety of tumour types, including KICH, LIHC, PAAD and UVM (Figure 5A). The findings imply that RBM39 may modulate the pattern of tumour immunity in some cancers by changing the expression levels of these immune checkpoint genes. We tallied the number of neoantigens in each tumour type and looked at the relationship between RBM39 expression and the number of neoantigens (Figure 5B). The expression of RBM39 in LGG was shown to be positively linked with the count of neoantigens (*R* = 0.158, *p* = 0.027), and in BRCA, the expression of RBM39 was negatively correlated with the number of neoantigens (*R* = –0.147, *p* < 0.001).

### 3.8 Relationship between the RBM39 expression and tumour immune cell infiltration

Next, we investigated the association of RBM39 expression with the level of infiltration of 24 immune‐related cells. Our data showed that the level of immune cell infiltration was substantially linked with RBM39 expression in the majority of malignancies (Table S1). We screened eight tumours for further analysis, including BRCA, COAD, HNSC, LIHC, LUSC, SKCM, STAD and UCEC, for which the correlation between RBM39 expression and the level of immune cell infiltration was high (Table 1). In the eight tumours analysed, the expression level of RBM39 correlated with B cells, Dendritic cells(DC) and their subpopulations aDC, iDC, pDC; Eosinophils, Macrophages, Mast cells, Neutrophils, Natural killer cells (NK cells) and their subpopulations: CD56+ NK cells, CD56 NK cells; T lymphocytes (T cells), CD8+ T cells, T helper cells(Th cells) and their subpopulations: Th1 cells, Th2 cells, Th17 cells; T cell regulatory(Tregs) and their subpopulations of fractionated cells: Tcm, Tem, TFH, Tgd, and Cytotoxic cells. As seen from the table, the correlation of RBM39 expression with B cells, DC and their subpopulations iDC and pDC, macrophages, neutrophils, CD56 NK cells, T cells, Th cells, and cytotoxic cells was negative consistently. In contrast, the expression of RBM39 in these eight tumours showed a positive correlation with both Th cells and the subpopulation Tcm of Tregs. In other immune‐infiltrating cells, these eight cells showed different correlations. For example, RBM39 positively correlates with aDC in LIHC, while it negatively correlates with aDC in the other seven tumours.

**TABLE 1:** Relationship between RBM39 expression and immune cell infiltration in different cancers

Cell type	BRCA	COAD	HNSC	LIHC	LUSC	SKCM	STAD	UCEC
	(*p*‐value/Cor)	(*p*‐value/Cor)	(*p*‐value/Cor)	(*p*‐value/Cor)	(*p*‐value/Cor)	(*p*‐value/Cor)	(*p*‐value/Cor)	(*p*‐value/Cor)
B cells	<0.001/−0.220	0.001/−0.150	0.059/−0.084	0.645/−0.024	<0.001/−0.280	0.070/−0.084	<0.001/−0.210	<0.001/−0.180
Dendritic cells	<0.001/−0.270	<0.001/−0.300	<0.001/−0.310	<0.001/−0.360	<0.001/−0.380	<0.001/−0.360	<0.001/−0.300	<0.001/−0.300
aDC	<0.001/−0.220	<0.001/−0.320	0.002/−0.130	0.265/0.058	<0.001/−0.240	<0.001/−0.180	0.043/−0.100	0.008/−0.110
iDC	<0.001/−0.130	<0.001/−0.370	<0.001/−0.320	0.071/−0.093	<0.001/−0.430	<0.001/−0.340	<0.001/−0.290	<0.001/−0.400
pDC	<0.001/−0.150	0.014/−0.110	<0.001/−0.210	<0.001/−0.290	<0.001/−0.170	<0.001/−0.340	<0.001/−0.420	<0.001/−0.420
Eosinophils	<0.001/0.230	0.425/−0.036	0.001/−0.150	0.001/0.170	<0.001/−0.160	0.081/−0.080	0.045/−0.100	0.187/−0.056
Macrophages	<0.001/−0.270	<0.001/−0.210	<0.001/−0.190	0.830/−0.011	<0.001/−0.360	<0.001/−0.210	<0.001/−0.220	0.120/−0.066
Mast cell	<0.001/0.110	<0.001/−0.220	<0.001/−0.290	0.062/−0.096	<0.001/−0.330	<0.001/−0.300	<0.001/−0.360	<0.001/−0.150
Neutrophils	<0.001/−0.240	<0.001/−0.220	<0.001/−0.420	<0.001/−0.260	<0.001/−0.390	<0.001/−0.380	<0.001/−0.210	<0.001/−0.330
NK cells	0.024/0.068	<0.001/−0.190	0.852/−0.008	0.432/−0.041	0.778/0.013	<0.001/−0.290	<0.001/−0.260	<0.001/−0.220
NK CD56bright cells	<0.001/0.130	<0.001/−0.380	0.010/0.120	0.016/0.130	0.044/0.090	<0.001/−0.290	0.765/−0.015	<0.001/−0.370
NK CD56dim cells	<0.001/−0.280	<0.001/−0.190	<0.001/−0.220	0.040/−0.110	<0.001/−0.230	<0.001/−0.280	<0.001/−0.190	<0.001/−0.330
T cells	<0.001/−0.150	<0.001/−0.330	<0.001/−0.160	0.398/−0.044	<0.001/−0.270	0.001/−0.150	<0.001/−0.210	<0.001/−0.250
CD8 T cells	0.008/−0.080	0.015/−0.110	0.815/0.010	0.517/−0.034	0.002/−0.140	0.022/0.110	<0.001/−0.240	0.054/−0.082
T helper cells	<0.001/0.320	<0.001/0.250	<0.001/0.240	<0.001/0.430	0.001/0.140	<0.001/0.440	<0.001/0.360	<0.001/0.390
T cells regulatory (Tregs)	<0.001/−0.250	<0.001/−0.280	<0.001/−0.170	0.007/−0.140	<0.001/−0.280	<0.001/−0.360	0.006/−0.140	<0.001/−0.360
Tcm	<0.001/0.280	<0.001/0.370	<0.001/0.170	<0.001/0.200	<0.001/0.230	<0.001/0.430	<0.001/0.320	<0.001/0.470
Tem	0.015/−0.073	0.109/−0.073	0.430/−0.035	0.718/0.019	0.569/0.025	0.182/−0.062	0.508/0.034	<0.001/−0.150
TFH	0.075/−0.054	<0.001/−0.190	0.001/−0.150	<0.001/0.200	0.001/−0.150	<0.001/−0.220	0.001/−0.170	<0.001/−0.200
Tgd	0.029/−0.066	0.802/0.011	0.005/−0.120	<0.001/−0.200	<0.001/−0.160	<0.001/0.250	0.711/−0.019	0.324/0.042
Th1 cells	<0.001/−0.240	<0.001/−0.270	<0.001/−0.190	0.519/0.033	<0.001/−0.340	<0.001/−0.170	0.002/−0.160	0.011/−0.110
Th2 cells	0.121/−0.047	0.004/−0.130	0.112/0.071	<0.001/0.340	<0.001/−0.180	0.005/0.130	0.057/0.098	<0.001/0.170
Th17 cells	0.769/0.009	0.027/−0.100	<0.001/−0.210	0.469/−0.038	<0.001/−0.160	<0.001/−0.320	0.027/−0.110	<0.001/−0.160
Cytotoxic cells	<0.001/−0.270	<0.001/−0.390	<0.001/−0.190	<0.001/−0.310	<0.001/−0.240	<0.001/−0.230	<0.001/−0.280	<0.001/−0.330

In addition, there was a differential relationship between RBM39 expression and different subpopulations of T cells. The results showed that RBM39 was negatively correlated with T cells and Tregs, whereas positively correlated with Th cells and Tcm. Tumours with the strongest correlation coefficients between the degree of infiltration of each immune cell and RBM39 expression was shown in Figure S6.

**FIGURE 6:**
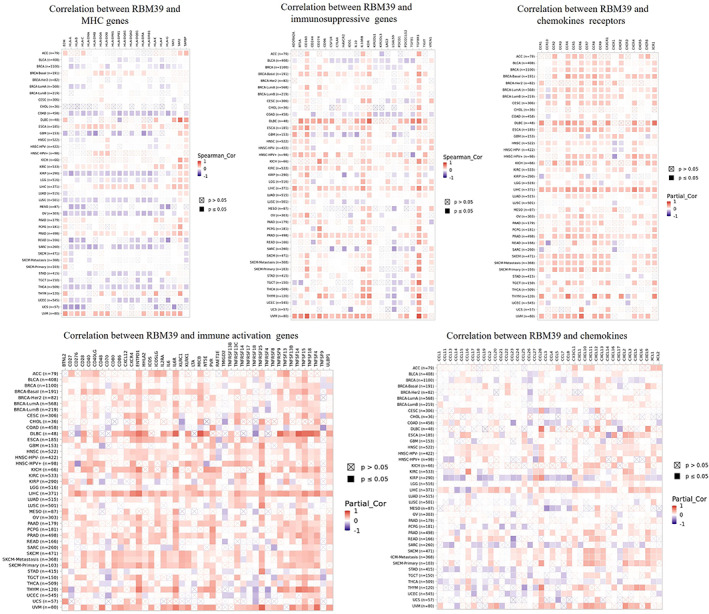
Co‐expression of RBM39 and immune‐related genes

We investigated the relationship between RBM39 expression and immune‐related genes in 33 malignancies using gene co‐expression analysis. Genes encoded MHC, immune activation, immune suppression, chemokine and chemokine receptor proteins were analysed. Heat maps showed positive correlations of RBM39 with immune‐related genes in KIRC, LIHC, PRAD and UVM (Figure 6).

### 3.9 GSEA enrichment analysis

**FIGURE 7:**
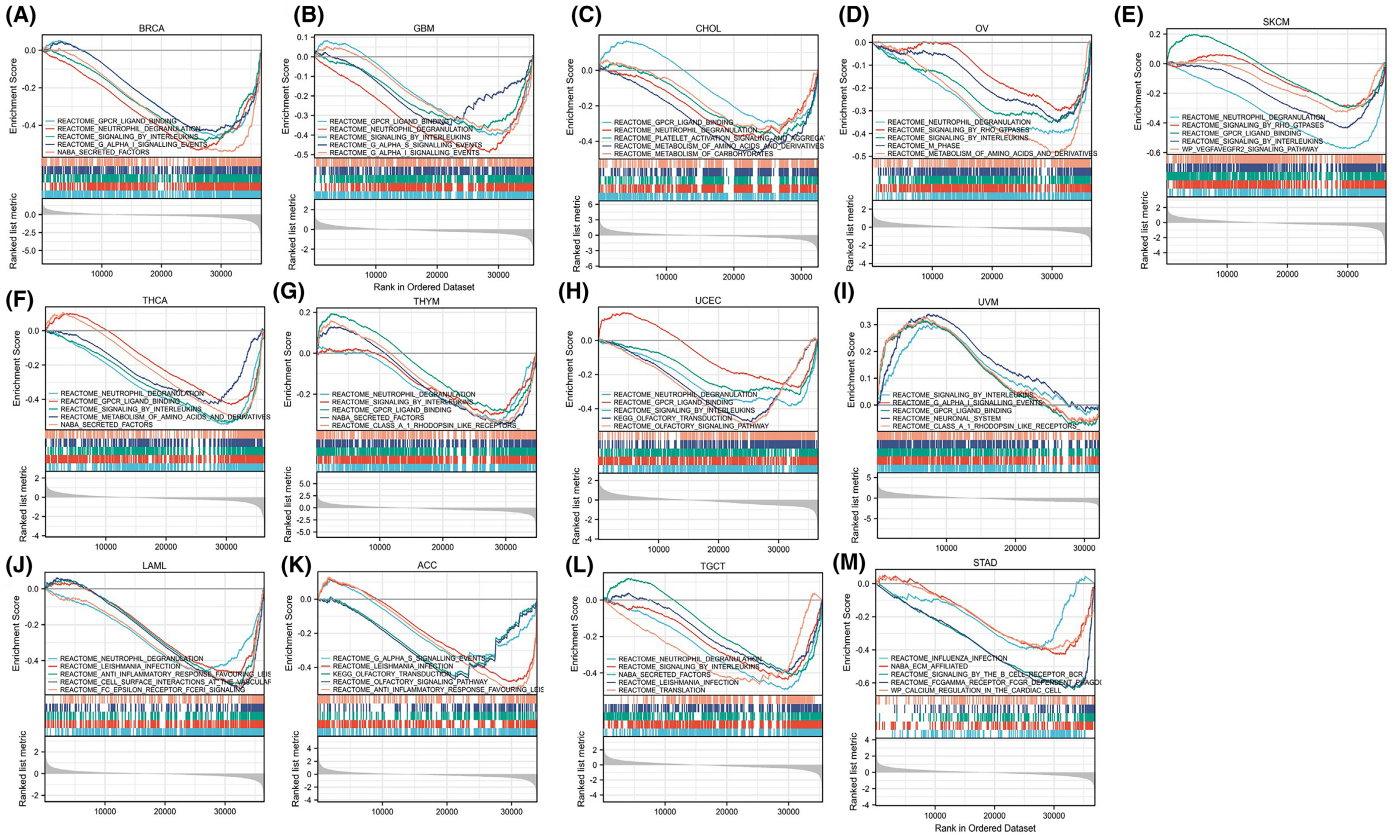
Results of GSEA

We used GSEA enrichment analysis to investigate the biological importance of RBM39 expression in various tumour tissues. The data showed that in nine tumours, including BRCA, GBM, CHOL, OV, SKCM, THCA, THYM, UCEC and UVM, RBM39 expression negatively correlates with immune‐related functions such as neutrophil degranulation and chemokine transduction (Figure 7A–I). While in LAML, ACC, TGCT and STAD, RBM39 expression negatively regulates anti‐infection responses, including leishmaniasis, common cold and so on (Figure 7J–M).

## 4 DISCUSSION

An increasing amount of evidence reveals that RBM39 is substantially linked to cancer growth in a range of malignancies. As an RNA‐binding protein, RBM39 is extensively involved in selective splicing of RNA.^14^It has been demonstrated that RBM39 is essential for RNA splicing in neuroblastoma cells.^15^ Of the 27 tumours we studied in the GTEx and TCGA databases, the RBM39 gene was highly expressed in six cancers and lowly expressed in 19 tumours. Until now, there have been some studies on the relationship between RBM39 and tumours. For example, Mercier I et al.^16^ showed that RBM39 was not detected or expressed at low levels in normal breast tissue with a cytoplasmic localization. In contrast, it was expressed at high levels in breast ductal carcinoma in situ specimens and was mainly distributed in the nucleus. RBM39 is a coactivator of ER, ER, and Activator Protein‐1 (AP‐1) component c‐Jun, which binds transcription and mRNA precursor processing together and effectively boosts their transcriptional activity, hence encouraging breast cancer growth.^17^ Another study reported that RBM39 acts as a major transcriptional regulator and interacts with the MLL1 complex to promote breast cancer cell proliferation.^18^ In patients with hepatocellular carcinoma, RBM39 was first identified as an autoantigen whose overexpression reduced tumour angiogenesis and growth and inhibited v‐Rel‐mediated lymphocyte transformation.^19^, ^20^, ^21^ Previous studies have shown that RBM39 is involved in biological processes in colorectal adenocarcinoma development, such as cell survival and anchoring non‐dependent growth.^22^

Using TCGA data, we performed a Kaplan–Meier survival analysis and discovered that greater RBM39 expression was associated with a poor prognosis for OS, DSS, and PFI in LIHC. RBM39 was discovered as an autoantigen in a cirrhotic patient who later acquired hepatocellular cancer.^23^ These findings suggest that RBM39 may be an oncogene during liver carcinogenesis. We also found that RBM39 expression was associated with age in certain types of cancer. Our study showed that RBM39 expression was lower in older patients in HNSC, KIRP and SARC, while in UCEC patients, reduced expression of RBM39 was associated with younger age. These results may have important implications for guiding the selection of immunotherapy regimens for patients of different ages. Our study also found that the expression of RBM39 in most cancers correlated with tumour stage, and the difference in expression was particularly significant in stage I and II tumours. For example, RBM39 expression was significantly lower in stages I and II of KICH. These findings imply that RBM39 might be employed as a biomarker to predict the prognosis of different malignancies.

Tumour mutational burden is an intriguing predictive biomarker with the potential to usher immuno‐oncology into the era of precision medicine.^24^ Previous studies have shown that in melanoma, whole‐exome sequencing (WES) of TMB is a predictor of increased survival in patients treated with ipilimumab or tremelimumab.^25^ Another study used TMB as a biomarker in non‐small cell lung cancer patients receiving pembrolizumab and WES and found an overall response rate (ORR), progression‐free survival (PFS) and long‐term therapeutic benefit in patients with a high somatic nonsynonymous mutation burden.^26^ Thus, tumours with higher TMB are assumed to have more neoantigens that can be recognized by the immune system in response to immune checkpoint inhibition. MSI is also a useful biomarker for immune checkpoint inhibitors (ICI). MSI in tumour DNA is defined as the presence of alternate‐sized repetitive DNA sequences that are not seen in the corresponding germline DNA. Depending on the type and the number of microsatellites analysed, widely variable results on the frequency of MSI in different tumour types have been published.^27^, ^28^ It has been shown that MSI is the first DNA marker that can be used to identify hereditary colorectal cancer.^29^ Our study showed that RBM39 expression correlated with TMB in 9 cancer types and with MSI in 12 cancer types. This might imply that RBM39 expression levels influence tumour TMB and MSI, and consequently the patient's response to immune checkpoint inhibition medication. This would give a new basis for immunotherapy prognosis. In addition, we also investigated the correlation of RBM39 expression with five MMRs and four DNA methyltransferases.

Our results suggest that RBM39 plays an important role in cancer immunity. The tumour microenvironment (TME) not only influences tumorigenesis, development and metastasis, but also has a profound impact on therapeutic results.^30^ According to the assessment scores, RBM39 expression in TME of lung squamous cell carcinoma only was negatively correlated with both mesenchymal and immune cell content. Furthermore, previous studies have shown that RBM39 is a proto‐oncogene that plays an important role in the development and progression of several types of malignancies. Changes in RNA splicing inside cancer cells produce neoepitopes, which are used as neoantigens in immunodetection site‐blocking investigations.^31^ RBM39 is considered as a novel human tumour‐associated antigen, and its specific immunity has been reported in a variety of tumours.^32^ We analysed the relationship between RBM39 expression and the number of neoantigens in 19 tumours. We found that RBM39 was positively correlated with the number of neoantigens in LGG, and negatively correlated with the number of neoantigens in BRCA. A major advance in cancer treatment is the development of immune checkpoint inhibitors (ICIs)^33^. Our results also showed that RBM39 expression was positively correlated with the expression levels of immune checkpoint genes in four types of cancer, suggesting that RBM39 may regulate tumour immune patterns through the number of tumours neoantigens and by regulating the expression levels of immune checkpoint genes. It has been demonstrated that when the interaction between checkpoint ligands and their cognate receptors on effector cells is blocked, a robust and long‐lasting antitumor response can be observed.^34^, ^35^

Tumour‐infiltrating immune cells have an important impact on tumorigenesis and progression and can antagonize or promote tumorigenesis and progression.^36^ The presence of certain immune subgroups usually exhibits a favourable prognostic effect in a malignancy.^37^ Recent studies have found that T lymphocyte subsets predict response to existing and emerging immunotherapies, highlighting the importance of studying tumour‐associated immune cells as potential predictive biomarkers.^38^, ^39^ Previous studies have reported that immunomodulatory drugs, such as sulfonamides, can recruit the splicing factor RBM39 to the E3 ligase substrate receptor DCAF15, leading to ubiquitination and degradation of RBM39, which resulted in altered RNA splicing and death in some cancer cell lines.^40^, ^41^, ^42^, ^43^ Moreover, RBM39 expression plays a role in intracellular immunosuppression. It has been shown that RBM39 orchestrates a splicing program that is essential for the survival of AML cells according to CRISPR/Cas9 screens.^44^, ^45^ Thomas R et al.^46^ have demonstrated that RBM39 is a critical RBP required for AML survival and that RBM39 deficiency causes leukaemia progression to be slowed and overall survival to be improved. Jia Tong et al.^47^ have found that RBM39 was overexpressed in myeloma cells and associated with poor prognosis of patients, and they demonstrated that the α‐DARS‐AS1‐RBM39 axis of HIF‐1 is a potential target for the treatment of multiple myeloma.

Our study further elucidates the broader tumorigenic applicability of RBM39 and confirms that RBM39 expression is closely associated with the biological processes of immune cells and immune‐related molecules in most tumours. In addition, our study revealed that RBM39 is co‐expressed with genes encoding MHC, immune activation, immune suppression, chemokine and chemokine receptor proteins. These results suggest that RBM39 expression is closely associated with immune infiltration of tumour cells, affecting patient prognosis and providing a new target for immunosuppressant development. Furthermore, our enrichment analysis suggests that RBM39 may influence the aetiology or pathogenesis of cancer by acting in immune cell infiltration, immune chemokine transduction and immune anti‐infection pathways.

In conclusion, our first pan‐cancer RBM39 analysis revealed that it is differentially expressed in tumour and normal tissues and that there is a link between RBM39 expression and clinical prognosis. Our findings imply that RBM39 can function as an independent prognostic biomarker in a wide range of malignancies. RBM39 expression leads to different prognostic outcomes for different tumours, which requires further investigation of the specific role of RBM39 in each tumour. In addition, RBM39 expression is associated with the tumour microenvironment and immune cell infiltration in different cancer types. Its effect on tumour immunity also varies by tumour type. These findings may help to elucidate the role of RBM39 in tumour development and provide a reference for achieving more precise and personalized immunotherapy in the future.

## AUTHOR CONTRIBUTIONS

**Rui Zhang:** Conceptualization (equal); data curation (equal); formal analysis (equal); investigation (equal); methodology (equal); writing – original draft (equal); writing – review and editing (equal). **Wei Wang:** Data curation (equal); investigation (equal); writing – review and editing (equal). **Xueting Chen:** Formal analysis (supporting); writing – review and editing (supporting). **Nie Zhang:** Formal analysis (supporting); writing – review and editing (supporting). **Wanming Liu:** Formal analysis (supporting); writing – review and editing (supporting). **Longzhen Zhang:** Funding acquisition (supporting); project administration (supporting); writing – review and editing (lead). **Nianli Liu:** Conceptualization (equal); funding acquisition (lead); project administration (lead); supervision (lead); writing – review and editing (lead).

## CONFLICT OF INTEREST

The authors report no conflicts of interest in this work.

## Supporting information


Figure S1

Figure S2

Figure S3

Figure S4

Figure S5

Figure S6

Table S1

## Data Availability

The data that supports the findings of this study are available in the supplementary material of this article.
