# Supplementary figures and images for: Systematic pan‐cancer analysis identifies RBM39 as an immunological and prognostic biomarker
Source: J Cell Mol Med. 2022 Aug 21;26(18):4859–71. doi: 10.1111/jcmm.17517 (PMC9465192; doi:10.1111/jcmm.17517)

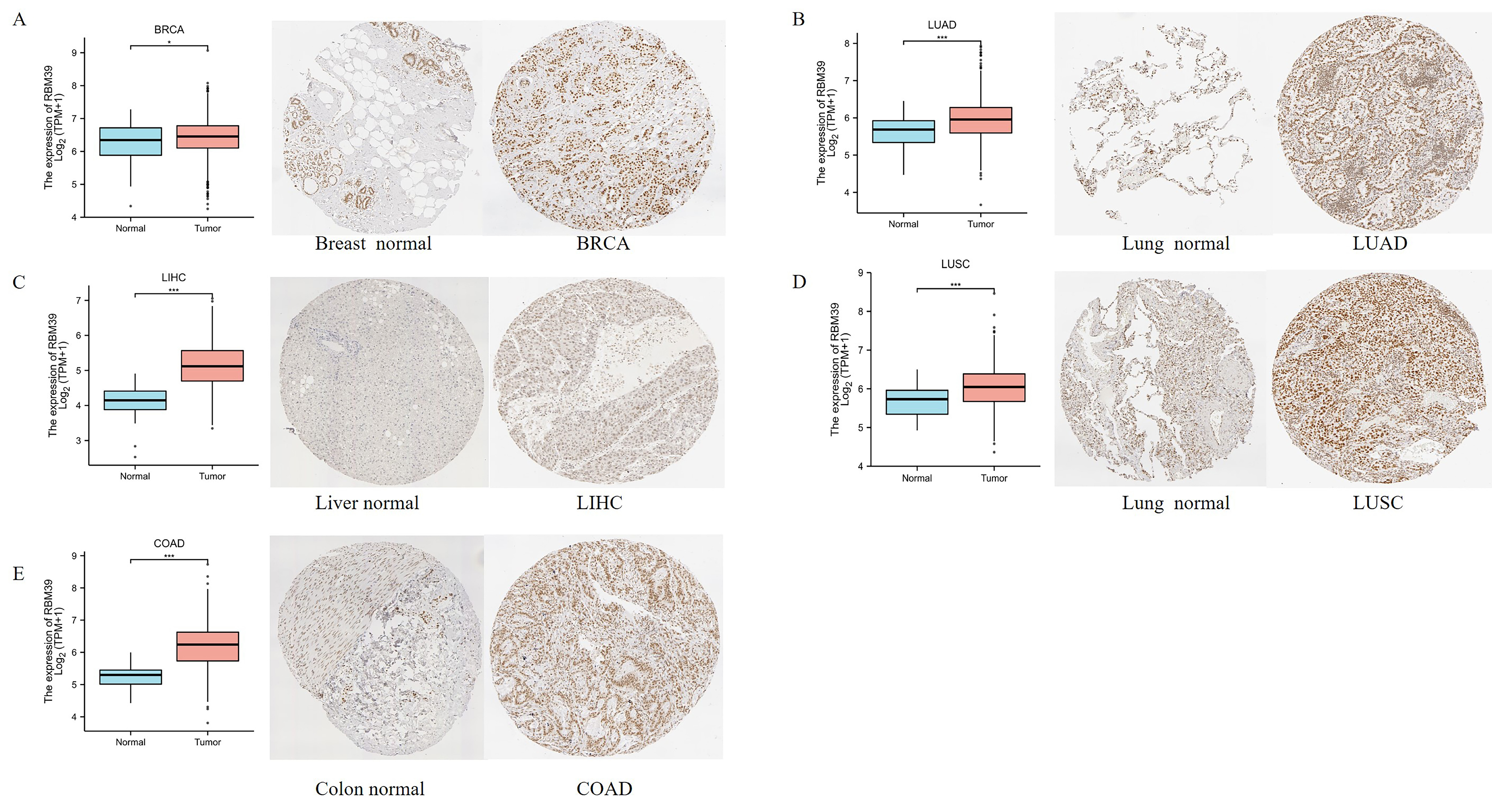

Supplement: Supplementary file 1 — Figure S1 [file JCMM-26-4859-s003.jpg]

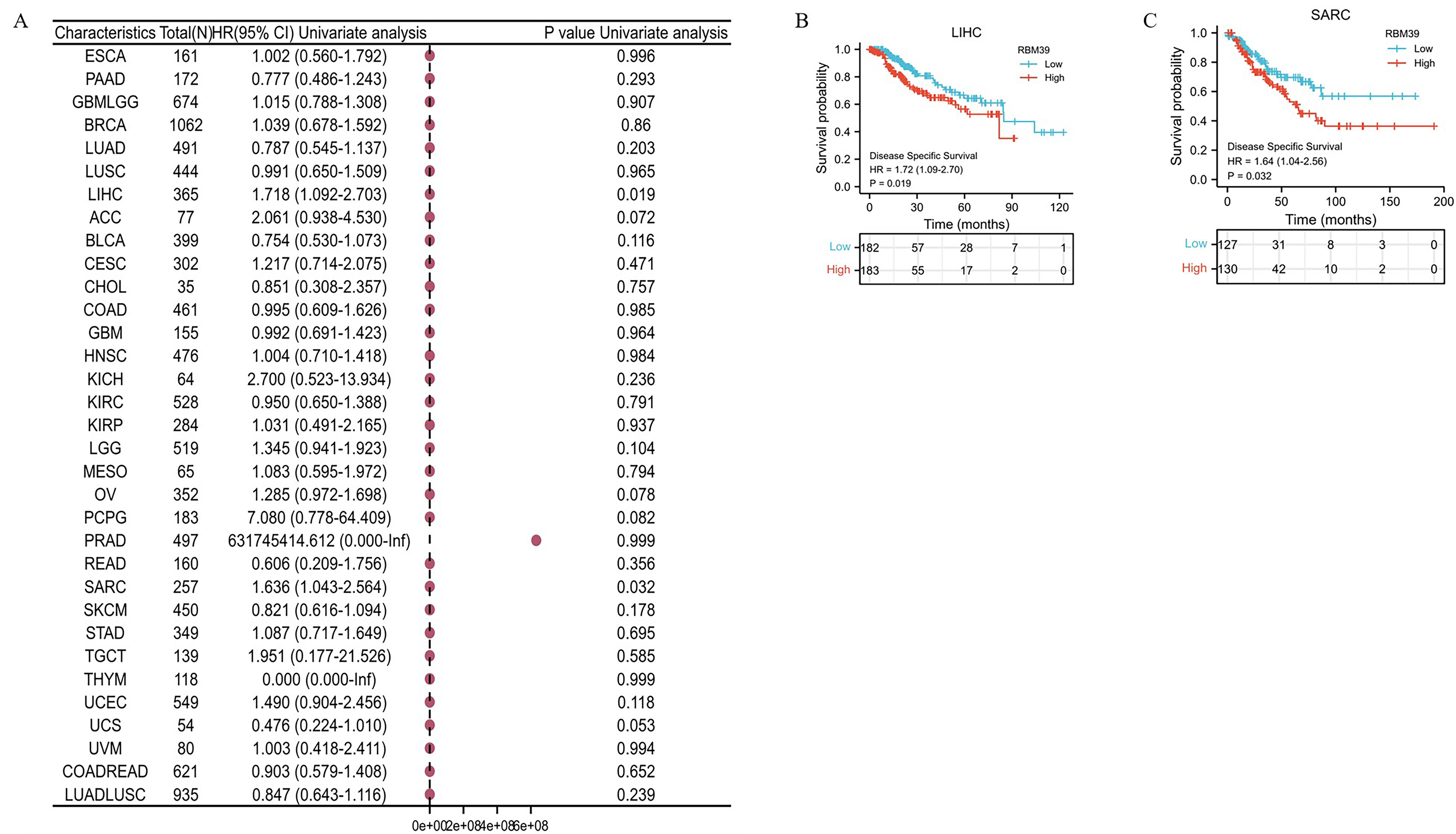

Supplement: Supplementary file 2 — Figure S2 [file JCMM-26-4859-s004.jpg]

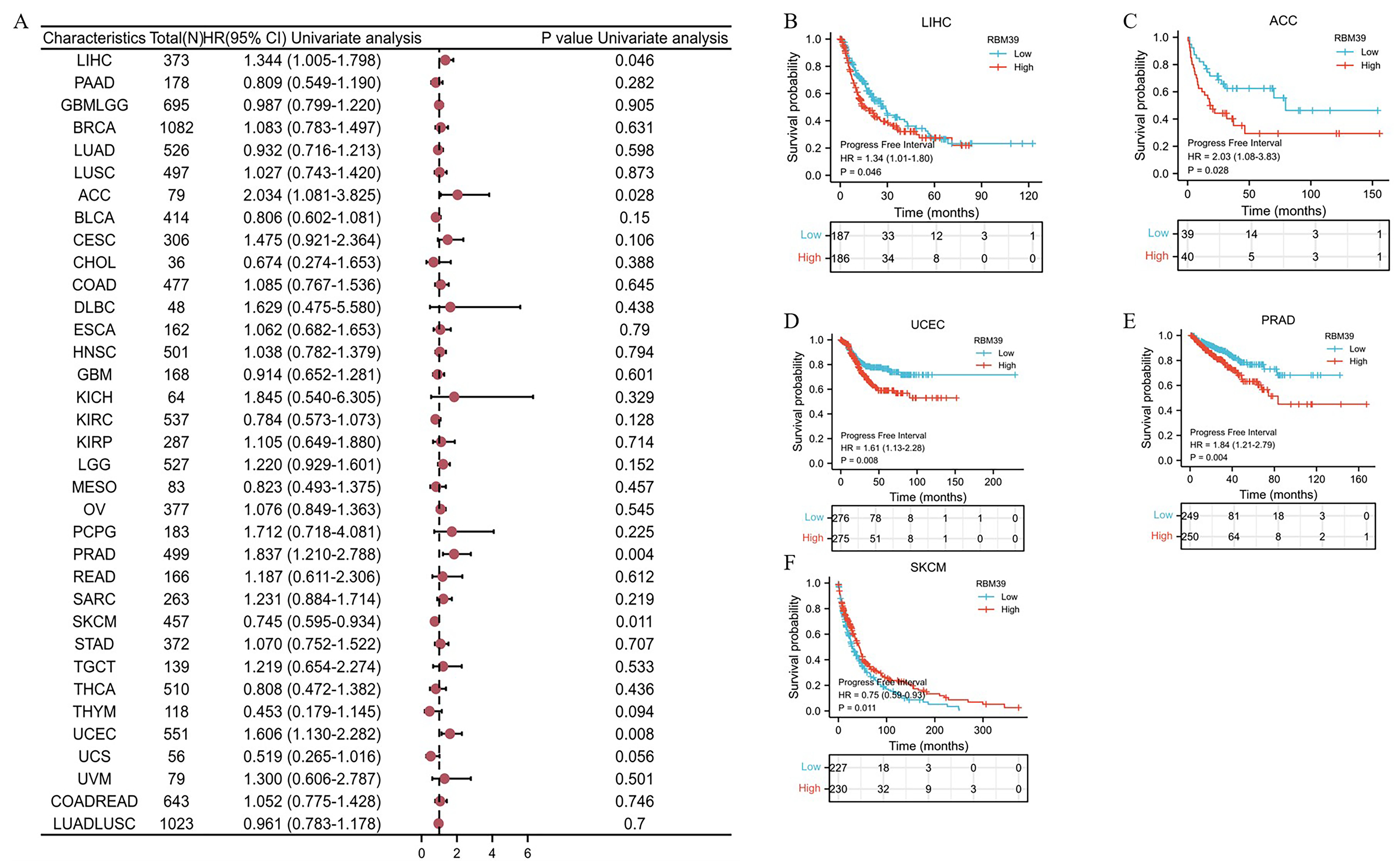

Supplement: Supplementary file 3 — Figure S3 [file JCMM-26-4859-s006.jpg]

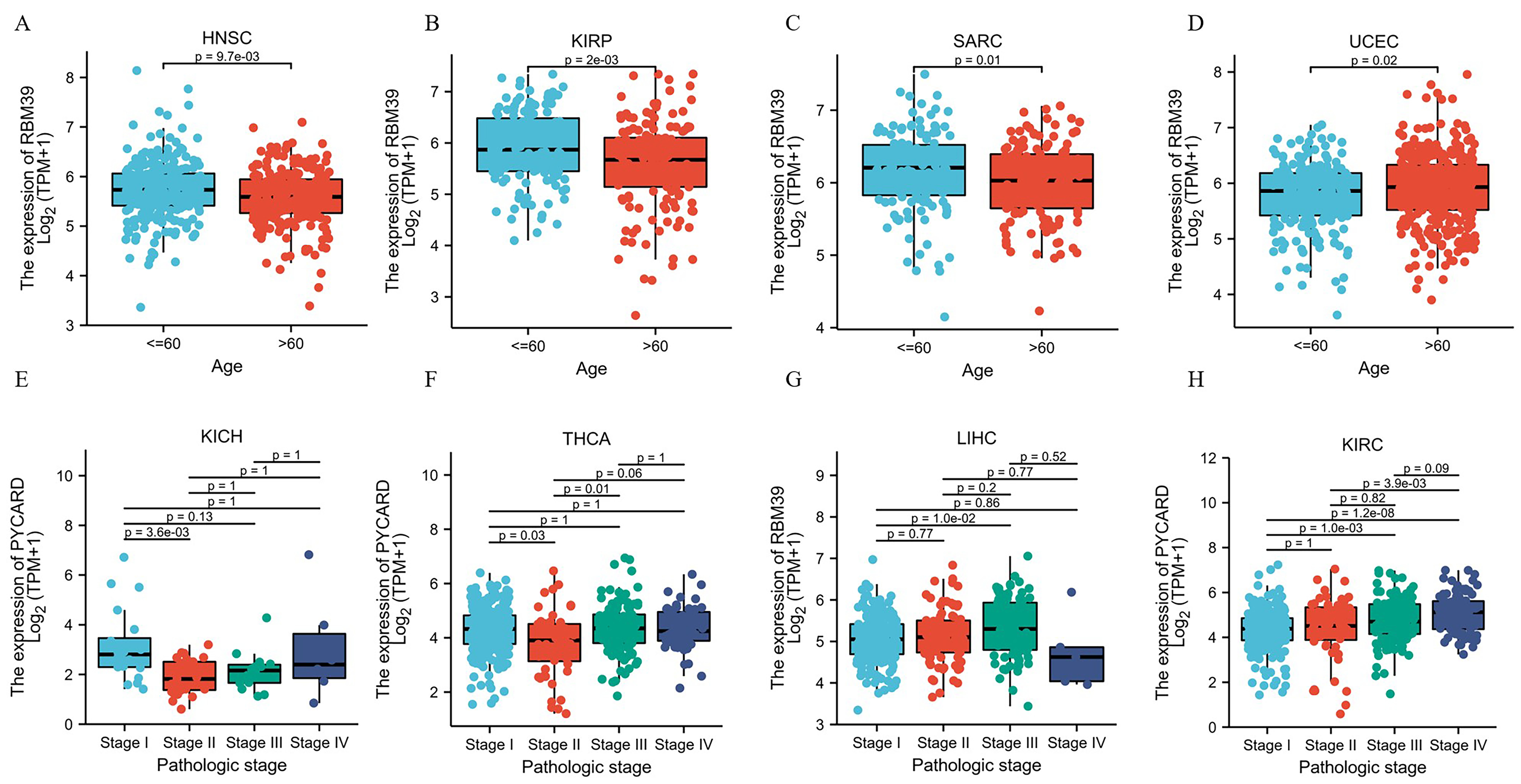

Supplement: Supplementary file 4 — Figure S4 [file JCMM-26-4859-s001.jpg]

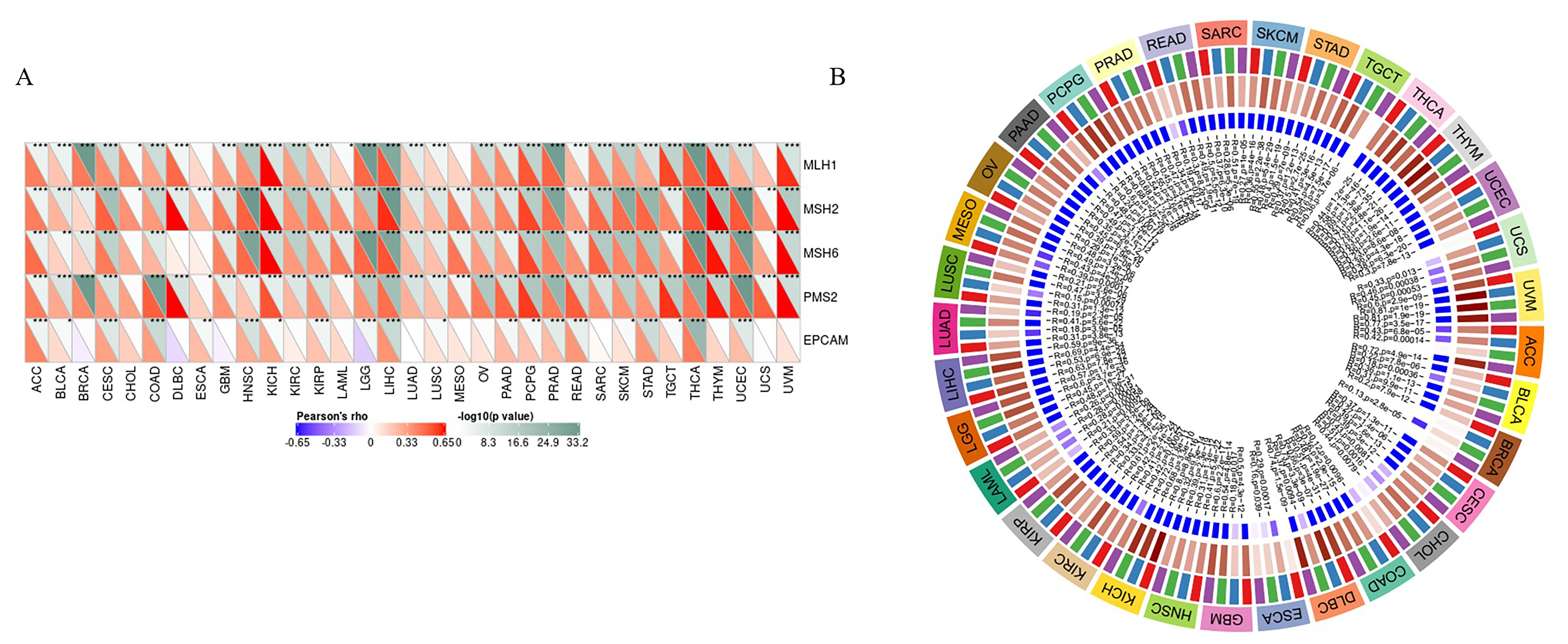

Supplement: Supplementary file 5 — Figure S5 [file JCMM-26-4859-s007.jpg]

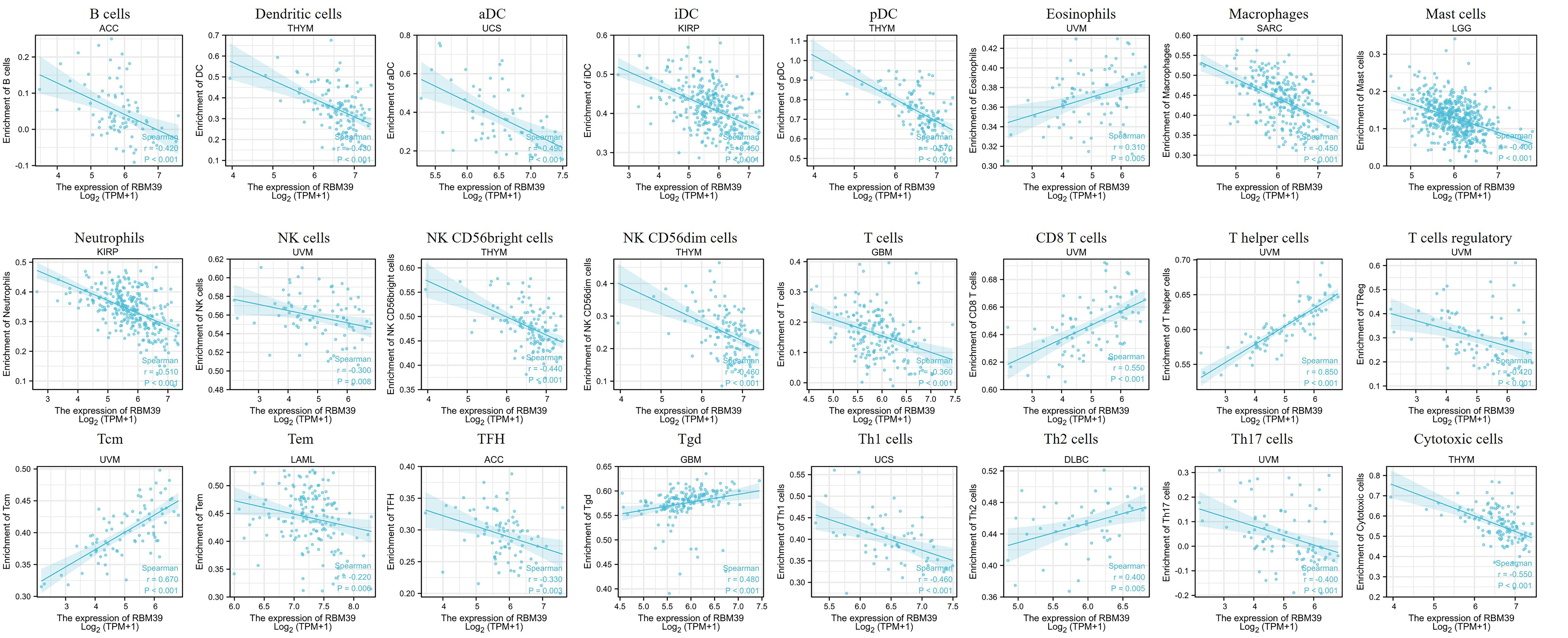

Supplement: Supplementary file 6 — Figure S6 [file JCMM-26-4859-s005.jpg]
